# Ready, set, differentiate!

**DOI:** 10.7554/eLife.01839

**Published:** 2013-12-17

**Authors:** Margarida Sancho, Tristan A Rodríguez

**Affiliations:** 1 **Margarida Sancho** is in the British Heart Foundation Centre for Research Excellence, National Heart and Lung Institute, Imperial Centre for Translational and Experimental Medicine, Imperial College, London, United Kingdom; 2 **Tristan A Rodríguez** is in the British Heart Foundation Centre for Research Excellence, National Heart and Lung Institute, Imperial Centre for Translational and Experimental Medicine, Imperial College, London, United Kingdomtristan.rodriguez@imperial.ac.uk

**Keywords:** BMP, Id1, Pluripotent stem cells, Cdh1, Mouse

## Abstract

The expression of E-Cadherin, a protein best known for its role in cell adhesion, regulates the onset of embryonic differentiation.

**Related research article** Malaguti M, Nistor PA, Blin G, Pegg A, Zhou X, Lowell S. 2013. Bone morphogenic protein signalling suppresses differentiation of pluripotent cells by maintaining expression of E-Cadherin. *eLife* **2**:e01197. doi: 10.7554/eLife.01197

**Image** Down-regulation of the protein E-Cadherin is required for embryonic stem cells to start the process of becoming neural cells, and thus express specific neural markers (marked in green)

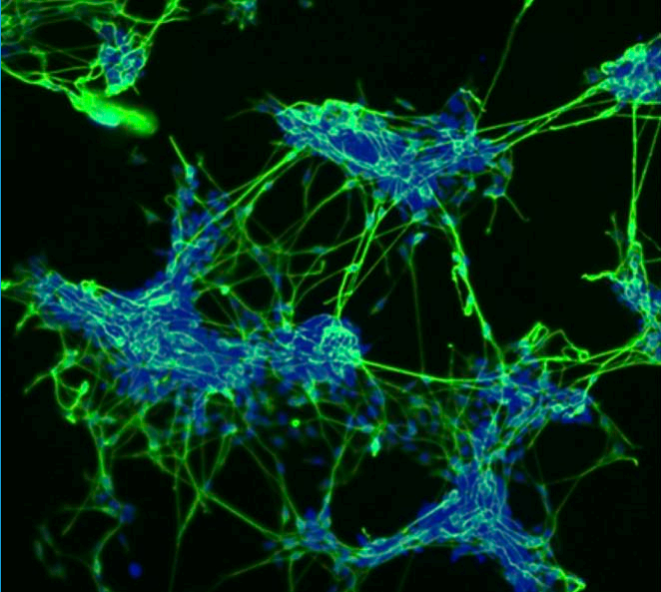

In mammals, during the early stages of development, the embryonic cells are similar and have the ability to become all the different cell types of the foetus—a property known as pluripotency. As such, a number of questions have fascinated cell and developmental biologists right from the birth of their fields. How and when do these embryonic stem cells stop being pluripotent and start to become different from each other? And how do they commit to becoming a specific type of cell?

To date, research has focussed on the signalling molecules, transcription factors and epigenetic processes that control this transition. Now, in *eLife*, Sally Lowell and colleagues at the University of Edinburgh—including Mattias Malaguti as the first author—supply a new twist to this story. They find that exposing pluripotent cells to different signals will pre-determine their fate: however, the process of differentiation can only begin once the expression of E-cadherin, a protein widely known for its role in cell adhesion, has been down-regulated (Malaguti et al., 2013).

The BMP pathway—where BMP is short for bone morphogenic protein—has been widely studied by developmental biologists, and evidence from a wide variety of model organisms, including frogs and mice, shows that BMPs prevent pluripotent cells from becoming neural cells during early development (Vonica and Brivanlou, 2006; Di-Gregorio et al., 2007). Furthermore, in mice, this is achieved via the up-regulation of genes that code for Id proteins (Ying et al., 2003). The BMP pathway is also involved in the formation and development of the mesoderm—the layer of cells in the early embryo that differentiate to become muscles, connective tissues and some blood cells (Kishigami and Mishina, 2005). However, we know very little about the mechanisms through which BMP is involved in all these processes.

Malaguti et al. have tackled this problem by analysing which cells respond to BMPs in early mouse embryos. They found that *Id1,* a gene up-regulated in response to BMP signalling, is activated in those cells that lie at the posterior of the embryo and that are about to form the mesoderm: however, this gene is not activated within the mesoderm itself. Furthermore, treatment of embryonic stem cells with BMPs during the first stages of their differentiation did not necessarily commit these cells to the mesoderm. Rather, it led them to express molecular markers that are characteristic of epithelial, mesodermal and pluripotent cells, all at the same time. Therefore, rather than inducing mesoderm differentiation per se, BMP seems to instead ‘prime’ pluripotent cells, making them ready for this change.

The observation that BMP caused pluripotent cells to remain epithelial by sustaining expression of E-Cadherin led Malaguti et al. to hypothesize that this protein could be the key factor downstream of BMPs in pluripotency. This hypothesis is intriguing as it suggests that E-Cadherin is involved (along with BMPs) in inhibiting the differentiation of stem cells into neural cells. At a first look this would seem unlikely because although E-Cadherin has been implicated in the regulation of pluripotency (Chou et al., 2008; Faunes et al., 2013; Livigni et al., 2013), it is best known as a protein involved in cell adhesion. The down-regulation of E-Cadherin is key for ‘epithelial to mesenchymal transitions’ in the early embryo: these transitions allow future mesoderm cells to detach from their pluripotent neighbours and move to form the mesoderm (Nieto, 2013). Importantly, no such transition has been described during neural differentiation.

However, Malaguti et al. challenged this view by showing that, when pluripotent cells differentiate into neural lineages, the expression of genes associated with pluripotency declined in concert with E-Cadherin expression. Further, the inhibition of E-Cadherin accelerated and increased the efficiency of this differentiation. This argues that down-regulation of E-Cadherin promotes neural differentiation, but does it mean that the main role of BMP signalling in pluripotency is to maintain E-Cadherin expression? This question was answered by Malaguti et al. with an elegant experiment which tested the effect of different levels of BMPs in cells where E-Cadherin has been inhibited. They found that low levels of BMP could not block neural differentiation when E-Cadherin is blocked, but high levels of BMP drove the cells to a primed mesoderm fate independent of E-Cadherin. Therefore BMP signalling has at least two separate roles in pluripotent cells: to repress neural identity by maintaining E-Cadherin expression, and to prime them for mesoderm formation.

These findings raise some fundamental questions: For example, what is the role of E-Cadherin in pluripotent stem cells? And how does its down-regulation lead to differentiation? To start to answer these questions it is important to consider the series of events that leads to cells becoming destined to become neural tissue. In the mouse embryo, when the future neural cells lose BMP signalling, they start to express molecular markers that are characteristic of the future brain whilst remaining pluripotent (Di-Gregorio et al., 2007; Osorno et al., 2012) and continuing to express E-Cadherin (Malaguti et al., 2013). This suggests that a cell’s first step in committing to a neural fate is likely to occur after the loss of BMP signalling and before the down-regulation of E-Cadherin. As such, E-Cadherin appears to allow pluripotent cells to remain in a state of dual identity for a short time: during this time the fates of the cells have essentially been determined, but they still have the potential to choose other fates.

It is tempting to speculate that by keeping cells pluripotent while the first step of commitment occurs, E-Cadherin ensures that, within a population of stem cells, all cells respond to a signalling molecule in a coordinated fashion. This would prevent the first cells that see the signal from ‘jumping the gun’ and initiating differentiation without their neighbours. In this way the cells in the early embryo would first respond to the different levels of BMP signalling: high levels of BMP would prime pluripotent cells to become part of the mesoderm, and low BMP levels would encourage differentiation into neuronal cells. Subsequently, when the levels of E-Cadherin are down-regulated, all the cells would lose their pluripotency and start to differentiate into their pre-destined fate in a co-ordinated manner (Figure 1).

**Figure 1.**
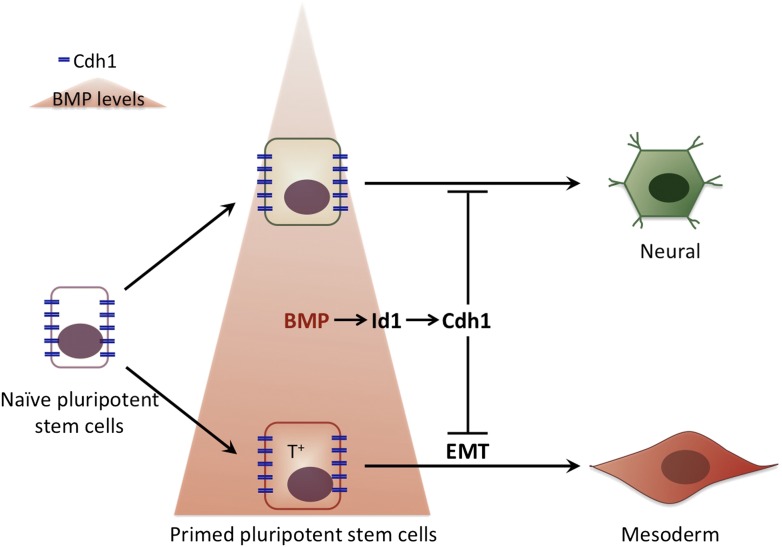
The influence of bone morphogenic protein (BMP) on cell fate choice. Pluripotent cells can become many different types of cells so they have to ‘choose’ their fate at some point. Malaguti et al. suggest that exposure to different levels of BMPs can pre-determine this choice: low levels of BMP (top) prime the pluripotent cells to become neural cells, whereas high BMP levels (bottom) prime the pluripotent cells to become part of the mesoderm. The expression of E-Cadherin (Cdh1) in response to BMP signalling maintains cells that are already committed to a particular fate in a pluripotent stage: however, cells only differentiate to adopt this fate once E-Cadherin has been down-regulated. The cells that are primed to become part of the mesoderm express a specific molecular marker (T+) and undergo an epithelial to mesenchymal transition (EMT) as they differentiate.

Moreover, it was recently shown that embryonic stem cells can also form the types of cells that are found in the extra-embryonic tissues, namely the placenta and yolk-sac (Morgani et al., 2013). This raises another question: could it be that E-Cadherin is determining the sensitivity of cells to developmental signals in the first cell fate choices that occur during mammalian development, namely those that determine an embryonic or an extra-embryonic fate?
